# Structural and Mechanical Properties of Ti–Co Alloys Treated by High Pressure Torsion

**DOI:** 10.3390/ma12030426

**Published:** 2019-01-30

**Authors:** Boris B. Straumal, Anna Korneva, Askar R. Kilmametov, Lidia Lityńska-Dobrzyńska, Alena S. Gornakova, Robert Chulist, Mikhail I. Karpov, Paweł Zięba

**Affiliations:** 1 Institute of Solid State Physics and Chernogolovka Scientific Center, Russian Academy of Sciences, Chernogolovka 142432, Russia; Askar.Kilmametov@kit.edu (A.R.K.); alenahas@issp.ac.ru (A.S.G.); Karpov@issp.ac.ru (M.I.K.); 2 Karlsruhe Institute of Technology (KIT), Institute of Nanotechnology, 76344 Eggenstein-Leopoldshafen, Germany; 3 National University of Science and Technology «MISIS», Moscow 119049, Russia; 4 Institute of Metallurgy and Materials Science, Polish Academy of Sciences, 30-059 Krakow, Poland; a.korniewa@imim.pl (A.K.); L.Litynska@imim.pl (L.L.-D.); R.Chulist@imim.pl (R.C.); p.zieba@imim.pl (P.Z.)

**Keywords:** titanium alloys, high pressure torsion, microhardness

## Abstract

The microstructure and properties of titanium-based alloys can be tailored using severe plastic deformation. The structure and microhardness of Ti–4 wt.% Co alloy have been studied after preliminary annealing and following high pressure torsion (HPT). The Ti–4 wt.% Co alloy has been annealed at 400, 500, and 600 °C, i.e., below the temperature of eutectoid transformation in the Ti–4 wt.% Co system. The amount of Co dissolved in α-Ti increased with increasing annealing temperature. HPT led to the transformation of α-Ti in ω-Ti. After HPT, the amount of ω-phase in the sample annealed at 400 °C was about 80­85%, i.e., higher than in pure titanium (about 40%). However, with increasing temperature of pre-annealing, the portion of ω-phase decreased (60–65% at 500 °C and about 5% at 600 °C). The microhardness of all investigated samples increased with increasing temperature of pre-annealing.

## 1. Introduction

Titanium and its alloys possess low density, high strength, as well as high corrosion resistance in the broad temperature interval. Titanium alloys were found to have broad applications in the aircraft, building, and medicinal industries. Due to their outstanding biocompatibility, Ti-alloys are increasingly applied in orthopaedic and dental implants. But titanium alloys don’t only have advantages. Unfortunately, the high melting temperature, high elastic modulus, and high affinity for oxygen can limit their application as biomaterials [1,2]. Fortunately, the structure and properties of titanium alloys can be tailored using various combinations of thermal and mechanical treatments. One of the promising new options is the so-called severe plastic deformation (SPD). SPD permits to rich the extremely high strains in a material without its failure. Disadvantages of titanium and its alloys can also be improved by the addition of alloying elements like niobium, zirconium, hafnium, molybdenum, cobalt, and chromium [2,3].

At the focus of this work will be Ti–Co alloys subjected to high pressure torsion (HPT), being one of the SPD modes. The Ti–Co alloys are broadly used as implant alloys in dentistry and medicine for many years [4,5,6,7,8]. Thus, the Ti-based alloys with cobalt addition show higher strength [9,10] and have lower melting temperature, which can alleviate many casting problems. The addition of cobalt improves the corrosion resistance of titanium [11] and its mechanical properties [12]. The ternary (Ti–Co)-based alloys also found broad applications [13,14,15,16,17,18]. The Ti–Co alloys are frequently used as coatings on other titanium alloys like Ti6Al4V [19,20,21,22,23]. Such surface modifications permit improvement of the endurance of Ti6Al4V alloy due to the formation of hard Ti-Co intermetallic particles. The Ti–Co thin films were used also as diffusion barriers, or as an element of integrated circuits [24,25].

SPD not only refines the grains of metallic alloys (including those of titanium) [26,27,28]. SPD also drives bulk and grain-boundary phase transformations [29,30,31,32,33]. In titanium these are the transitions between the low-temperature α-phase, high-temperature β-phase, and high-pressure ω-phase [34,35,36,37,38,39]. The high-pressure ω-phase appears in Ti-based alloys during HPT and then retains after pressure release [26,27,40,41]. Previously, it has been studied how ω-phase transforms during HPT from the mixture of α- and β-phases [26,42,43]. It has been observed that β-to-ω transformation goes along quite easily [26,43,44]. It is martensitic, follows a special orientation relationship, and does not need intensive mass transfer. However, the HPT-driven β-to-ω transformation in Ti-4 wt.% Co alloy proceeds less easily in comparison to the Ti-4 wt.% Fe one [44]. Most probably, the reason is the less favorable coincidence of lattice constants between β- and ω-phases in the Ti-4 wt.% Co alloy. The α-to-ω transformation in Ti-based alloys encounter more troubles than β-to-ω [26,43]. Mainly it is because the orientation relationship between α and ω phases is less favorable [26,34,35,36,37,38,39,44]. How would the high-pressure ω-phase form in the case of only α-phase and intermetallic precipitates existing in a sample before HPT? In order to answer this question, we studied the properties of Ti–4 wt.% Co alloy where the HPT of the α + β mixture had already been investigated [19,43,44,45]. We annealed the Ti–4 wt.% Co alloy for extremely long durations below eutectoid temperature in order to produce the α-Ti solid solution with a different (and equilibrium) concentration of cobalt, as well as a different amount of possible coarse Ti_2_Co precipitates.

## 2. Experimental

**Figure 1:**
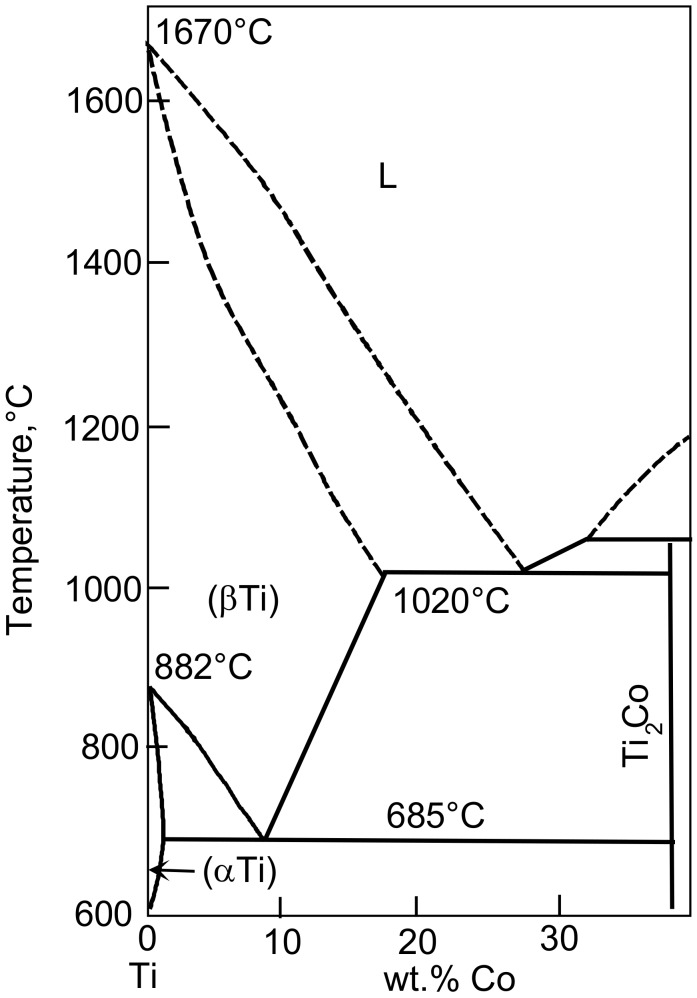
The Co-rich part of the Ti–Co phase diagram [46].

For the preparation of Ti–4 wt.% Co alloys, pure titanium (99.98%) and cobalt (99.99%) were been used. The concentration of 4 wt.% Co was on the left side of the point of eutectoid β → α + Ti_2_Co transformation (8.5 wt.% Co, see Figure 1). The alloy was melted in the argon atmosphere with the aid of an induction furnace and cast into ingots cylindrical with a diameter of 10 mm. The resulting ingots were spark erosion cut into 0.7 mm thick disks. The resulted slices were chemically etched and put into the ampoules. The residual pressure in the sealed quartz ampoules was about 4 × 10^−4^ Pa. The annealing temperatures were 400, 500, and 600 °C, i.e., below the temperature of eutectoid transformation in the Ti–Co system. We annealed the ampoules during a very long period (for 5685, 5685, and 2774 h, respectively) in order to reach the equilibrium cobalt content in the αTi-based solid solution. The ampoules with samples inside were quenched in cold water after annealing. Then, the ampoules were broken and disks were treated at room temperature with the aid of HPT in a Bridgman anvil type unit using a custom built computer-controlled device (W. Klement GmbH, Lang, Austria) with 5 plunger rotations. The strain rate was 1 rpm, the pressure was 7 GPa, and the thickness of the samples after HPT was 0.35 mm.

Measurements of the microhardness were performed using the PMT-3 unit with the load of 20 g. The samples were carefully polished before measurements with 1 μm diamond paste. We measured microhardness at least 10 times for each sample at the distance from the disk center of about half of its radius [45]. The Siemens D-500 X-ray diffractometer with Cu-Kα radiation was used for the investigations of X-ray diffraction (XRD). The software PowderCell for Windows Version 2.4.08.03.2000 (Werner Kraus & Gert Nolze, BAM Berlin) allowed us to calculate the lattice parameters and to perform the phase analysis. The FEI E-SEM XL30 SEM (Hillsboro, OR, USA) equipped with EDAX Genesis energy-dispersive X-ray spectrometer (EDS) permitted us to conduct the scanning electron microscopy (SEM) investigations. The TECNAI G2 FEG super TWIN (200 kV) TEM (Hillsboro, OR, USA) operating at an accelerating voltage of 200 kV was used for the transmission electron microscopy (TEM) studies. The TEM instrument was equipped with an energy dispersive X-ray (EDS) spectrometer manufactured by EDAX. We prepared thin foils for TEM using an electrolyte D2 manufactured by Struers company (Cleveland, OH, USA). 

## 3. Results and Discussion

Figure 1 shows part of the Ti–Co phase diagram (with low cobalt content) [46]. The sample composition of Ti–4 wt.% Co is on the left side of the point of eutectoid β → α + Ti_2_Co transformation (8.5 wt.% Co). The annealing temperatures were 400, 500, and 600 °C, and they are located below the temperature of eutectoid transformation *T*_e_ = 685 °C. The maximum solubility of cobalt in α-Ti is about 1.2 wt.% Co and is reached at *T*_e_ = 685 °C.

**Figure 2:**
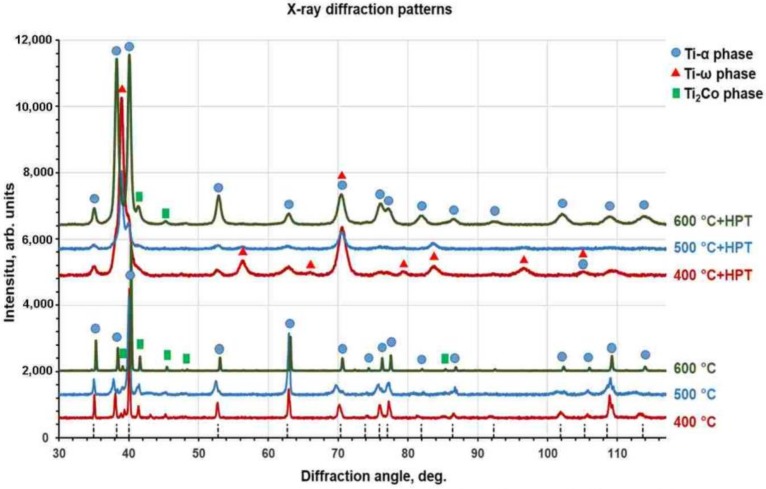
X-ray diffraction patterns for Ti–4 wt.% Co alloy after annealing at 400, 500 and 600 °C (lower patterns) and after high pressure torsion (HPT) with preliminary heat treatment (upper patterns). Vertical dotted lines show the positions of the reflections for pure α-Ti.

In Figure 2, the X-ray diffraction patterns for Ti–4 wt.% Co alloy preliminary annealed at 400, 500, 600 °C (lower patterns), and for the same samples, but after following HPT (upper patterns) are shown. After annealing, all samples contained α-Ti and intermetallic compound Ti_2_Co. According to the phase diagram (Figure 1), the Ti_2_Co phase is daltonide and its composition does not change with the temperature. Therefore, the position of Ti_2_Co peaks in the X-ray diffraction patterns are the same for all three annealing temperatures. However, the amount of Ti_2_Co phase slightly decreased with increasing annealing temperature (see Table 1). This is because the total amount of cobalt in the alloy remained constant, and the cobalt solubility in the α-Ti based solid solution increased when the annealing temperature approached the eutectoid one. According to the phase diagram (Figure 1), the solubility of cobalt in α-Ti-based solid solution increased with a temperature below *T*_e_ = 685 °C. The increase of Co content in α-Ti decreases the lattice parameter [44]. Indeed, we can see in lower patterns in Figure 2 that the α-Ti peaks in the sample annealed at 600 °C are shifted to the right in comparison to samples annealed at lower temperatures (it means the decrease of lattice parameter) with increasing temperature. Thus, the amount of cobalt dissolved in α-Ti increases with increasing temperature. Since the total amount of cobalt remains the same (4 wt.% Co), the cobalt atoms for α-Ti were taken from Ti_2_Co precipitates, and their amount slightly decreased with increasing temperature (see Table 1). After HPT, all peaks in XRD patterns were broadened and their intensity decreased. It marked the usual for SPD strong grain refinement. Moreover, the ω-Ti phase appeared in all samples. A certain amount of α-Ti phase remained. The intermetallic phase Ti_2_Co was also present. The lattice parameters for α-Ti and ω-Ti before and after HPT were given in Table 1. The lattice parameters of ω-Ti are less sensitive to the temperature of pre-annealing than those of α-Ti. The lattice parameter *a* of α-Ti phase increased after HPT in all studied samples. The lattice parameter *c* of α-Ti phase also increased in the sample pre-annealed at 600 °C and slightly decreased in samples pre-annealed at 400 and 500 °C

**Figure 3:**
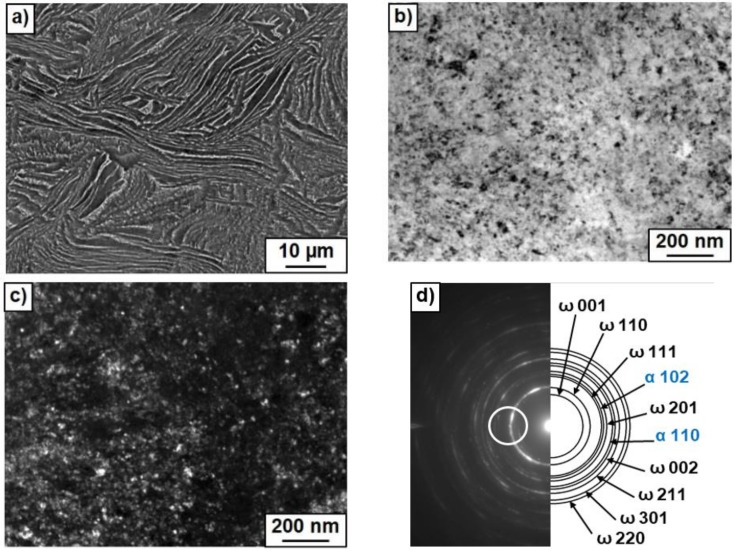
Microstructure of Ti–4 wt.% Co alloy after annealing at 400 °C and high pressure torsion (HPT). (**a**) Scanning electron microscopy (SEM) micrograph. (**b**) Bright field and (**c**) dark field transmission electron microscopy (TEM) micrographs after annealing at 400 °C and following HPT. (**d**) Selected area electron diffraction pattern.

**Figure 4:**
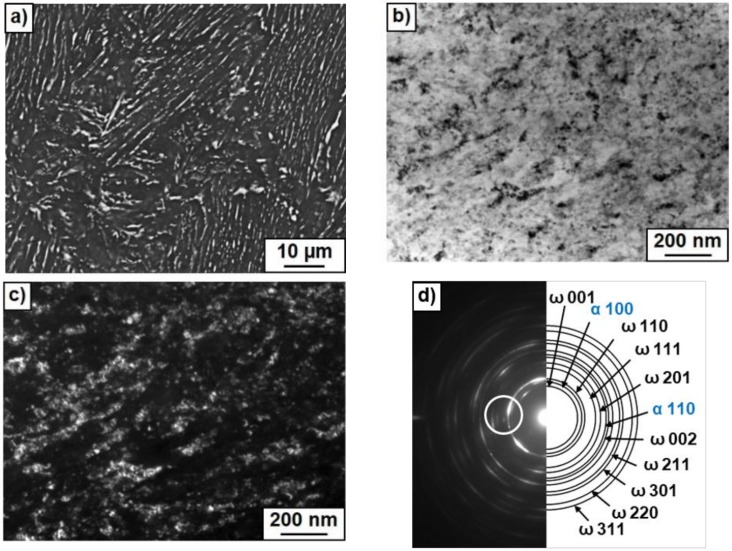
Microstructure of Ti–4 wt.% Co alloy after annealing at 500 °C and high pressure torsion (HPT). (**a**) Scanning electron microscopy (SEM) micrograph. (**b**) Bright field and (**c**) dark field transmission electron microscopy (TEM) micrographs after annealing at 400 °C and following HPT. (**d**) Selected area electron diffraction pattern.

**Figure 5:**
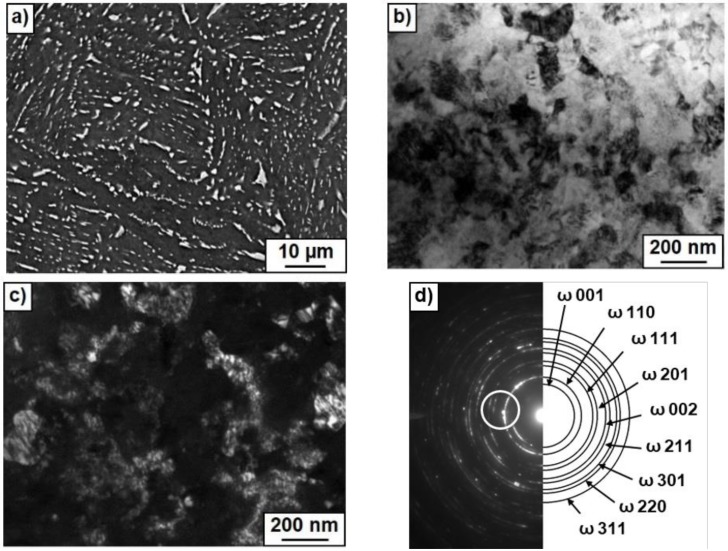
Microstructure of Ti–4 wt.% Co alloy after annealing at 600 °C and and high pressure torsion (HPT). (**a**) Scanning electron microscopy (SEM) micrograph. (**b**) Bright field and (**c**) dark field transmission electron microscopy (TEM) micrographs after annealing at 400 °C and following HPT. (**d**) Selected area electron diffraction pattern.

After HPT, the amount of ω-phase in the sample annealed at 400 °C was about 80–85%, i.e., higher than in pure titanium (about 40% [26]). However, with increasing temperature of pre-annealing the portion of ω-phase decreased (60–65% at 500 °C and about 5% at 600 °C). Earlier we observed that both Ti–Fe and Ti–Co alloys annealed above eutectoid temperature contain after HPT more ω-phase than the same HPT-treated alloys annealed before HPT below eutectoid temperature [44]. Also, the addition of aluminum to the binary Ti–V alloys completely suppressed the formation of (ωTi) phase after HPT [47]. The decrease of the amount of ω-phase with increasing temperature of pre-annealing can be indirectly driven by the change of the amount and morphology of intermetallic precipitates (see Table 1 and Figure 3, Figure 4 and Figure 5). 

Figure 3, Figure 4 and Figure 5 show the microstructure of Ti–4 wt.% Co alloy after annealing at different temperatures and HPT. Figure 3a, Figure 4a and Figure 5a show SEM micrographs. Figure 3b, Figure 4b and Figure 5b show bright field and Figure 3c, Figure 4c and Figure 5c dark field TEM micrographs after annealing and following HPT. Figure 3d, Figure 4d and Figure 5d show selected area electron diffraction patterns (SAED). The part of SAED used for DF images is shown by the circle. The main input to the DF images give the ω-100 ring. Therefore, the grains appearing bright in the DF images mainly represent the ω-phase. Particularly, it is visible how some ω-grains are elongated in the rotation direction of the HPT anvil. After HPT the grains of α-Ti and ω-Ti phases are very fine. Figure 3c,d, Figure 4c,d, and Figure 5c,d witness that the grain size of α-Ti and ω-Ti phases after HPT increased with increasing temperature of preliminary annealing (about 70 nm for 400 °C, 100 nm for 500 °C and 150 nm for 600 °C). It can be seen in SEM micrographs that the morphology of Ti_2_Co particles (they appear bright) is different after different temperatures of pre-annealing and HPT. With increasing temperature of pre-annealing, the Ti_2_Co particles (also after HPT) become bigger. It seems that the hard and coarse Ti_2_Co particles were less refined by HPT than the smaller ones. SAED-patterns witness that the samples contained at least two finely dispersed phases.

HPT changes of the lattice parameters *a* and *c* for α-Ti phase (Table 1). These changes are equivalent to the decrease of cobalt content in α-Ti phase [44]. This behavior is very similar to the recently observed “purification” of α-Ti phase in the Ti–Fe alloys after HPT [27]. 

Where can we move the cobalt atoms from the α-Ti phase during HPT? The first possibility is that they migrate into newly formed ω-Ti phase. It is known, for example, that the solubility of iron in ω-Ti is much higher than in the α-Ti phase [26]. We can suppose that a similar law is true for solubility of cobalt in ω-Ti and α-Ti. X-ray microanalysis in SEM mode indeed demonstrated that the areas predominately filled with ω-phase contained more cobalt than the areas predominately filled with α-phase. The second possibility is that the cobalt atoms are used to form the fine precipitates of Ti_2_Co phase. They are visible in the dark-field TEM images and contribute into SAED patterns. The third possibility are the grain boundaries (GBs) that additionally appear in the samples afterward HPT. In all materials subjected to HPT, the grain size decreased at least one order of magnitude [28,48,49,50,51,52,53,54,55,56]. In our case, the grains after HPT became almost a thousand times smaller. As a result, the GB area in the volume unit strongly increased. Cobalt atoms segregate in these new GBs. They are taken from the bulk solid solution. Due to this phenomenon of GB segregation, the overall (apparent) solubility of a second component strongly increased in nanograined materials [57]. Thus, the third reason for the “cleaning” of α-Ti phase during HPT is that the cobalt atoms are used to form the GB segregation. Similar HPT effect exists also in steels [58,59,60]. In steels also, only a few carbon atoms can be diluted in the α-Fe lattice. However, the GBs “help” to dissolve a large amount of carbon without formation of carbides [58,59,60]. During the HPT-driven “cleaning” of α-Ti phase in our experiments the cobalt atoms migrate from the volume solid solution to the GBs. Such HPT-driven atomic migration in Ti-alloys proceed very quickly [26,27]. The estimated equivalent diffusion coefficients of this diffusion-like mass transfer are several orders of magnitude higher than the coefficient of conventional volume diffusion extrapolated to the HPT temperature of 300 K [26]. This acceleration is especially astonishing because the applied pressure always decreases the rate of diffusion-controlled processes [61,62].

One can find in the published papers the mechanical properties of different Ti- phases [63,64,65]. The elastic moduli of α-Ti and β-Ti were determined in a Ti–4 wt.% V–6 wt.% Al alloy [63]. It appeared that was the elastic modulus of α-Ti in this coarse-grained alloy is 22% higher than that of the β-Ti. Moreover, the shear modulus of β-Ti in the samples annealed between 600 and 975 °C decreased with increasing temperature of annealing [64]. It was also shown theoretically that the specific energy of the α/β interphase boundary decreased with increasing temperature about two times [65].

**Figure 6:**
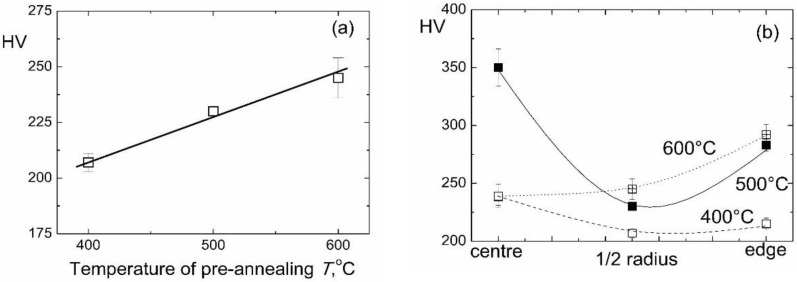
Dependence of microhardness of Ti–4 wt.% Co alloy after HPT on the temperature of preliminary annnealing (**a**) and on the position in the sample (**b**).

In Figure 6, the microhardness values were given for the HPT-treated samples after preliminary annealing. The microhardness measured in the middle of the radius increased from 210 to 250 HV with increasing temperature of pre-annealing (Figure 6a). We can suppose that this increase is due to the decrease of the portion of ω-phase in the samples. The hardness is influenced also by the hard Ti_2_Co intermetallic particles. The increase of the pre-annealing temperature slightly decreased the amount of Ti_2_Co particles (Table 1). They become larger (compare Figure 3a, Figure 4a and Figure 5a). These two facts would be the reason for the certain softening. On the other hand, the concentration of cobalt in α-Ti increased with the increasing temperature of pre-annealing (even after HPT). It can lead to a certain solid-solution hardening of the α-phase. The resulted influence of these three factors leads to the increase of microhardness. The increase of microhardness with an increase of the annealing temperature has been observed recently in Ti–V and Ti–V–Al alloys [47]. However, this similarity is superficial because the factors leading to the increase of microhardness in the Ti–V and Ti–V–Al alloys are most probably different. First, these alloys do not contain any intermetallic precipitates. Second, the low amount of ω-phase is present after HPT only in binary Ti–V alloys [47]. 

## 4. Conclusions

High pressure torsion leads to the phase transformations in the studied Ti–4 wt.% Co alloy. The samples were annealed below eutectoid temperature in order to produce the mixture of α-Ti phase with different cobalt concentrations and Ti_2_Co intermetallic precipitates. Thus, the initial phases before HPT were different from the previously studied α + β mixture. After HPT, the ω-Ti phase appeared in the samples. Its portion decreased with increasing temperature of pre-annealing. The microhardness of all investigated samples increased with increasing temperature of pre-annealing.

## Figures and Tables

**Table 1:** Phases, lattice parameter and their amount in studied titanium alloys after annealing and after following HPT.

Sample	Lattice Parameter, nm	Lattice Parameter, nm	Lattice Parameter, nm	Volume
-	Before HPT	After HPT	After HPT	Fraction, %
-	α-Ti	α-Ti	ω-Ti	ω-Ti
Ti–4 wt. %Co 600 °C, 2774 h	*a* = 0.2941, *c* = 0.4689, *c/a* = 1.594	*a* = 0.2957, *c* = 0.4703, *c/a* = 1.590	*a* = 0.4622, *c* = 0.2833	5
Ti–4 wt. %Co 500 °C, 5685 h	*a* = 0.2954, *c* = 0.4759, *c/a* = 1.611	*a* = 0.2966, *c* = 0.4718, *c/a* = 1.591	*a* = 0.4622, *c* = 0.2833	65
Ti–4 wt. %Co 400 °C, 5685 h	*a* = 0.2953, *c* = 0.4729, *c/a* = 1.602	*a* = 0.2963, *c* = 0.4725, *c/a* = 1.595	*a* = 0.4622, *c* = 0.2833	80
Pure Ti	*a* = 0.2953, *c* = 0.4694, *c/a* = 1.588	*a* = 0.2959, *c* = 0.4690, *c/a* = 1.585	*a* = 0.4627, *c* = 0.2830	40
